# Evaluation of cowpea mini core accessions for resistance to flower bud thrips *Megalurothrips sjostedti *Trybom (Thysanoptera: Thripidae)

**DOI:** 10.1111/jen.12637

**Published:** 2019-04-14

**Authors:** Abou Togola, Ousmane Boukar, Siva Chamarthi, Nouhoun Belko, Manuele Tamò, Nathaniel Oigiangbe, Joseph Ojo, Mumini Ibikunle, Christian Fatokun

**Affiliations:** ^1^ International Institute of Tropical Agriculture Kano Nigeria; ^2^ Africa Rice Center Bouaké Côte d'Ivoire; ^3^ International Institute of Tropical Agriculture Cotonou Benin; ^4^ Zoology Ambrose Alli University Ekpoma Nigeria; ^5^ International Institute of Tropical Agriculture Ibadan Nigeria

**Keywords:** cowpea genotypes, crop improvement, field screening, heritability, varietal resistance, yield loss

## Abstract

The flower bud thrips, *Megalurothrips sjostedti *Trybom (Thysanoptera: Thripidae), is an economically important pest of cowpea in sub‐Saharan Africa. Varietal resistance is the most preferred, environmentally friendly, cost‐effective and sustainable option for controlling this pest. The objective of this study was to identify sources of resistance to *M. sjostedti *among mini core accessions from the largest world cowpea germplasm collection maintained at the International Institute of Tropical Agriculture (IITA). The study was conducted during the 2015 and 2016 cropping seasons where 365 accessions were screened under field conditions. Each accession was rated visually for thrips damage score, flower abortion rate, number of pods per plant and number of thrips per flower. The resistance levels observed in genotypes TVu8631, TVu16368, TVu8671 and TVu7325 were similar to that of the resistant check “Sanzisabinli” (called Sanzi) during both seasons. In addition, 56 mini core genotypes showed moderate resistance to thrips damage. High heritability values were associated with thrips damage scores at 65 days after planting (0.60), percentage of effective peduncles (0.59), flower bud abortion rate (0.59), number of pods per plant (0.51) and number of peduncles with pods (0.5). The accessions identified with good levels of resistance to flower bud thrips will be used in cowpea breeding programs to develop improved resistant varieties.

## 1 INTRODUCTION

Cowpea (*Vigna unguiculata* (L.) Walp.; Fabaceae) is an important grain legume grown especially in the dry savannah agro‐ecologies of sub‐Saharan Africa (SSA). In Africa, cowpea is part of the traditional cropping systems and it is considered as a strategic crop because of its multiple uses as human food, animal feed, source of nitrogen for soil restoration and income for resource‐poor farmers as well as small‐scale processors (Abtew et al., 2016; Boukar, Fatokun, Huynh, Roberts, & Close, 2016; Isubikalu, Erbaugh, Semana, & Adipala, 2000). Cowpea grains represent a major source of protein (20%–32%), minerals and vitamins in the diet of majority of rural and semi‐urban communities (Boukar, Bhattacharjee, Fatokun, Kumar, & Gueye, 2013; Egho, 2010; Palanga et al., 2016; Singh, 2014). The tender leaves, soft stems and green pods are also eaten as vegetables in Asian and East African communities (Abudulai, Salifu, & Haruna, 2006). About 85% of the world cowpea production comes from the West Africa sub‐region (FAOSTAT, 2017). The International Institute of Tropical Agriculture (IITA) is conserving about 15,000 cultivated cowpea germplasm accessions from many countries in its Genetic Resources Centre.

Cowpea is an attractive host to many insect pests that reduce its grain yield and quality. Among them, the flower bud thrips, *Megalurothrips sjostedti *Trybom (Thysanoptera: Thripidae), causes the most serious damage during the crop's flowering stage (Jackai & Daoust, 1986). The insect lays eggs on cowpea flower buds and the nymphs/adults feed on the reproductive structures of the plant (Alabi, Odebiyi, Tamò, & Omoloye, 2011), resulting in drying out and browning of the stipules, flower bud abscission, flower discoloration, distortion or abortion (Jackai, Inang, & Nwobi, 1992; Kanteh, Ndoleh, Dimoh, & Luseni, 2013). Due to premature flower abortion, the peduncles of susceptible plants are stunted as no pods develop on them. Grain yield reduction due to the flower bud thrips ranges from 20% to 80% (Omo‐Ikerodah, Fawole, & Fatokun, 2008) and could reach 100% under high infestation (Alabi et al., 2011; Jackai & Daoust, 1986; Singh & Allen, 1980). Insecticide application is the predominant means of controlling this pest on cowpea. However, several alternative control measures including cultural practices (Ekesi, Maniania, & Ampong‐Nyarko, 1999), biological control (Ekesi & Maniania, 2000; Mfuti et al., 2017; Tamò, Ekesi, Maniania, & Cherry, 2003) and the use of bio‐pesticides such as neem extract (Badii, Nuamah, Braimah, & Awuku, 2016) have been explored to control this insect. Host plant resistance appears to be the most economical and environmentally friendly way to reduce thrips damage to cowpea. Unfortunately, most of the cowpea varieties grown in West Africa are highly susceptible to *M. sjostedti*. Only a few cowpea genotypes have been reported to show low levels of resistance to the pest (Omo‐Ikerodah et al., 2008). Hence, a systematic evaluation of the currently available germplasm accessions could lead to the identification of more lines with higher levels of resistance to this pest. This study was conducted to evaluate the cowpea mini core accessions from the world's largest collection maintained at the Genetic Resources Center of IITA for new sources of resistance/tolerance to flower bud thrips. The genomic tools that are being generated in cowpea (Muñoz‐Amatriaín et al., 2011) would further enhance the chances of successfully pyramiding these novel resistance genes in suitable varieties. Genotypes identified as thrips resistant will be used for the development of improved cowpea varieties, which would minimize the need for insecticide application by farmers when also ensuring increased grain yield.

## 2 MATERIALS AND METHODS

### 2.1 Study site

The study was conducted at Fashola, Latitude 7.9°N and Longitude 3.7833°E located between Oyo and Iseyin towns at about 60 km from Ibadan (Oyo State, Nigeria). The average temperature of the location during the main cropping season ranges from 21.42°C (*T*_min_) to 31.82°C (*T*_max_). The annual average relative humidity is 73.78%, while the annual average rainfall is about 1,173 mm. The soil type is Silt‐clay. The location has been identified as a hotspot for flower bud thrips from previous field experiments conducted by IITA Cowpea Breeding Unit.

### 2.2 Cowpea lines

The trials were carried out during 2015 and 2016 main cropping seasons (August–November) when cowpea is mostly grown by farmers in the area. A total of 369 cowpea germplasm accessions including 365 from the IITA mini core collection, one wild relative (TVNu699) and one Nigerian landrace (NGT65A) were screened under field conditions. A cowpea landrace from Ghana “Sanzisabinli” (called in short Sanzi) was used as resistant check because of its consistently low levels of damage scores and low population of thrips in flowers (Abudulai et al., 2006; Alabi et al., 2011; Sobda et al., 2018) while Vita 7 was used as susceptible check because of its well‐known high damage scores and high thrips populations in flowers (Abudulai et al., 2006; Omo‐Ikerodah et al., 2008).

### 2.3 Planting and experimental design

During both years, seeds of the susceptible check, Vita 7, were sown in single row along the four sides of each replicate two weeks prior to sowing of test lines to help build up the population of the flower bud thrips in the field. No chemical application was done in the experimental field during the pre‐flowering and flowering stages when cowpea is most susceptible to thrips. However, at podding stage, a synthetic pyrethroid insecticide, “Cyper‐Diforce EC,” composed of cypermethrin (30 g/L) and dimethoate (250 g/L) as active ingredients, was applied in the field to control other pests such as *Maruca vitrata* (Fabricius; Lepidoptera: Crambidae) and the pod sucking bugs complex. During both seasons, the cowpea test lines were planted following an Alpha Lattice Design with 20 blocks. The experimental plot size was 1.5 m × 2 m with 0.75 and 0.2 m plant spacing between and within rows, respectively. Each entry was sown in a single row within each block with two plants per hill. Distances of 0.75 and 1 m were left between consecutive plots and blocks, respectively. In both 2015 and 2016, all entries were planted in three replicates separated by a distance of 2 m. Two manual weedings were carried out using hoes during each season.

Thirty‐seven days after sowing the test lines, plants of the susceptible spreader Vita 7 were uprooted and laid down in between the rows of the test lines to allow the flower thrips to migrate to them.

### 2.4 Data collection

In 2015, thrips damage scores were recorded on each test line as exploratory data, while in 2016 additional parameters such as number of pods per plant, number of peduncles with and without pods, the flowering time, the stem/pod colour and the population of thrips were measured. The number of pods and number of peduncles were counted on five plants selected at random in each row. The flowering time was recorded as period when the first flower appeared on the plant. Three flowering groups were considered: early flowering (from planting up to 45 days), medium flowering (46 and 55 days) and late flowering (>56 days). The stem/pod colour was recorded at podding stage. Visual damage score ratings were recorded at three intervals. The first score was taken at 45 days after planting (DAP) when only a few test lines had flowered. The last two readings were recorded at 55 and 65 DAP (flowering/podding stage). The visual scoring was based on a 1–9 scale (Jackai & Singh, 1988), where 1 = no browning or drying of stipules and flower buds with no flower bud abscission; 3 = initiation of browning of stipules and/or flower buds but no flower bud abscission; 5 = distinct browning/drying of stipules and/or flower buds with some flower bud abscission; 7 = serious flower bud abscission accompanied by browning/drying of stipules and buds; and 9 = very severe flower bud abscission, heavy browning with drying of stipules and stunted peduncles. Number of pods and peduncles per plant were counted at plant maturity.

Based on the number of peduncles and pods, the percentage of effective peduncles (%Effpdcl) and the flower bud abortion rates (AR) were calculated as follows:

$$\%\;\text{Effpdcl}=\frac{\text{No}\;\text{peduncles}\;\text{with}\;\text{pods}}{\text{Total}\;\text{peduncle}\;\text{no}.}\times 100$$

$$\text{AR}=\frac{\text{No}\;\text{peduncles}\;\text{without}\;\text{pods}}{\text{Total}\;\text{peduncle}\;\text{no}.}\times 100$$

Thrips population was recorded on three flowers randomly collected per entry at 55 DAP. The flowers were collected between 8.0 and 10.0 a.m. and placed separately in labelled vials containing 70% ethanol and subsequently dissected in the laboratory to count the number of thrips (both adults and nymphs) per flower.

### 2.5 Definition of the resistance status

Based on the damage score ratings (Jackai & Singh, 1988), accessions with scores less than five (i.e., 1–4) were considered resistant while those with scores of five and seven to nine indicated moderate level of resistance and susceptible, respectively.

### 2.6 Statistical analysis

Data collected from the field and laboratory were subjected to analysis of variance using SAS 9.4 to determine if there were significant differences among the cowpea genotypes. The LSD test at 5% significance level was used to separate the means. A simple linear regression analysis was performed to show relationships between the damage score at 65 DAP (DS3) and selected variables of interest such as number of pods per plant (Pod‐plt), number of peduncles with pods (Pedclwpd), flower bud abortion rate (AR) and % effective peduncles (%Effpdcl). Breeding View software was used to establish the estimated correlations between all measured parameters namely damage scores (at 45 DAP, 55 DAP, 65 DAP), number of pods per plant, number of peduncles per plant, number of peduncles with pods, flower bud abortion rate, % effective peduncles and thrips population/flower. Also, the heritability values of these parameters were determined.

## 3 RESULTS

### 3.1 Thrips damage scores and population

There were significant differences in thrips damage scores among the cowpea lines tested at 45, 55 and 65 DAP during both years (Table 1). At 65 DAP, four genotypes (TVu8631, TVu16368, TVu8671 and TVu7325) showed consistently low damage scores in 2015 and 2016. Their damage scores ranged from 2.8 to 4.3 in 2015 and from 2.5 to 4.4 in 2016. The damage score rates of these genotypes were close to that of the resistant check, Sanzi. Also, 57 accessions showed moderate damage by *M. sjostedti*. Their damage scores ranged from 4.5 to 6.3. These genotypes were less resistant than Sanzi and less damaged by the insect when compared with Vita 7 (Table 1). No significant difference was noted among cowpea genotypes for the number of thrips per flower (Table 1). However, there was a relationship between damage scores and thrips population (Table 2).

**Table 1:** Means of measured parameters in the best cowpea test entries and the checks from 2015 and 2016 experiments

Var	Damage scores (DS) 2015			Damage scores (DS) 2016			Other measured parameters 2016					Status
	DS1	DS2	DS3	DS1	DS2	DS3	Pods/plt	% Effpdcl	Thrips Pop	Flow time	Stem/Pod colour	
Sanzi*	1.5	2.3	2.8	1.2	1.5	2.5	5.1	45.4	2.5	E	Purp	R
TVu16368	1.8	2.8	4.3	1.9	3.1	4.1	5.4	70.7	2.5	E	Green	R
TVu8631	2.8	3.3	4.0	2.3	3.0	4.2	6.5	48	1	E	Purp	R
TVu7325	2.0	3.3	4.3	2.2	3.1	3.8	5.7	49.5	1.5	L	Green	R
TVu8671	2.0	2.8	4.0	2.2	2.9	4.4	5.8	53.3	2.5	E	Green	R
TVu8877	2.0	2.8	4.0	1.7	2.5	4.4	3.7	59.8	0	M	Green	MR
TVu16521	2.8	4.0	5.5	2.3	2.9	4.4	4.2	56.6	0.5	E	Purp	MR
TVu7739	2.5	3.8	5.3	3.0	3.8	4.3	7	78.3	3.5	M	Green	MR
TVu8779	2.3	3.3	4.8	2.5	3.1	4.1	8.5	44.9	0.5	L	Green	MR
TVu9357	3.0	3.8	4.8	3.0	3.9	4.1	8.7	62.6	1	L	Green	MR
TVu4808	3.3	4.0	5.3	2.9	3.8	4.7	9.2	39.3	1.3	L	Green	MR
TVu9237	2.5	3.5	5.3	2.5	3.1	4.7	8.3	73.4	1	E	Green	MR
TVu7647	2.8	3.8	5.0	2.5	3.2	4.2	4.5	36.2	1.5	E	Green	MR
TVu6782	3.0	4.0	4.8	2.8	3.6	4.5	5.2	63.6	4.5	M	Green	MR
TVu7971	2.3	3.5	4.8	3	3.7	4.7	5.7	39.5	2.5	E	Purp	MR
TVu16403	2.0	3.0	4.0	2.3	4.7	5.3	6	42.5	2.5	M	Green	MR
TVu8612	2.8	3.3	5.0	2.2	3.5	4.7	3.8	45	1	L	Green	MR
TVu9265	3.5	4.8	6.0	3.0	3.8	4.8	5.2	55.6	1.5	M	Green	MR
TVu8834	2.8	3.3	3.8	2.6	3.8	4.9	5.1	43.7	0.5	E	Green	MR
TVu16528	3.3	3.8	5.3	3.3	4.7	4.7	5.6	39.7	0.5	M	Purp	MR
TVu15411	3.8	4.9	5.5	2.7	3.5	5	4.6	43.1	0	M	Green	MR
TVu7625	2.5	4.3	5.5	2.8	3.9	5.0	3.9	42.3	1.5	L	Green	MR
TVu8838	2.3	3.3	4.8	2.3	3.4	5.1	5.7	57.2	1	L	Green	MR
TVu15861	2.5	3.8	5.0	2.5	3.9	5.2	8.1	47.9	2.5	E	Green	MR
TVu16449	3.0	4.8	6.0	3.0	4.5	5.2	3.7	38.2	1	E	Green	MR
TVu7382	2.7	3.8	5.5	2.5	3.5	5.2	4.5	36.7	1.5	L	Green	MR
TVu7719	2.3	3.3	4.0	2.7	4.0	5.2	4.8	37.5	0	E	Green	MR
TVu8516	2.8	3.5	5.0	2.7	3.6	5.2	3.8	35.3	0	M	Green	MR
TVu16430	3.5	4.3	5.8	3.2	4.3	5.3	4.1	56.8	1	E	Green	MR
TVu9391	3.0	4.5	5.8	2.8	4.1	5.3	6.9	38.6	2.5	E	Purp	MR
TVu10466	4.0	5.0	5.8	3.4	4.5	5.3	5.8	38.5	13.5	M	Green	MR
TVu6464	3.5	4.0	5.3	2.6	3.6	5.6	3.6	38.2	1.5	E	Green	MR
TVu8656	2.5	3.8	5.5	2.4	3.4	5.4	6.2	78.1	0.5	E	Green	MR
TVu16504	2.8	3.8	5.0	2.3	3.8	5.7	6	69	0	M	Green	MR
TVu5307	3.5	4.3	5.5	2.7	4	5.3	8.7	35.5	3	M	Green	MR
TVu8812	2.8	3.8	5.0	2.3	3.3	5.7	5.2	36.7	0	L	Green	MR
TVu6837	3.5	4.8	6.0	2.3	2.7	5	5.1	38.3	0.5	E	Purp	MR
TVu8622	3.0	4.3	5.3	2.3	3	5.1	6.7	42	3	E	Green	MR
TVu16237	3.3	4.0	5.3	3.3	4.7	5.7	3.8	35.7	1.5	E	Green	MR
TVu16505	3.3	4.3	6.0	2.7	3.9	5.6	5	64.2	2.5	E	Purp	MR
TVu6477	2.5	3.3	4.5	2.8	3.7	5.6	2.6	36.4	3	E	Green	MR
TVu8262	3.3	4.0	5.5	3.3	4.7	5.6	3.8	35.9	0	M	Green	MR
TVu15878	3.3	3.5	4.8	2.6	3.4	5.7	5	34.2	0.5	E	Green	MR
TVu16486	4.0	5.5	6.3	3.4	4.3	5.7	3	34.3	1	E	Purp	MR
TVu415	3.0	4.0	5.3	2.4	4.2	5.7	4.5	42.2	2.5	M	Green	MR
TVu10408	2.5	3.5	4.8	2.8	3.4	5.7	3.5	34.9	1	E	Green	MR
TVu8702	2.3	3.5	5.3	2.3	3.4	5.7	4.6	39.7	1.5	E	Purp	MR
TVu6858	1.8	3.4	5.0	2.0	2.9	5.7	6.1	47.4	3.5	M	Green	MR
TVu14190	2.8	4.0	5.0	2.7	3.9	5.8	3.1	39.9	0	E	Green	MR
TVu53	3.5	4.8	5.5	2.8	4.1	5.8	3.9	33.5	0	M	Green	MR
TVu10754	2.8	4.0	5.3	2	3.5	6.3	4	38.3	0	L	Green	MR
TVu15445	3.5	4.5	5.8	2.7	4.3	6	2.9	41.8	1	M	Green	MR
TVu8775	3.8	4.8	6.3	2.9	3.8	5.9	2.8	69.8	1	M	Green	MR
TVu10179	3.0	4.0	6.0	4	4.7	6	4.2	46.6	4	M	Green	MR
TVu7798	3.3	4.0	5.5	2.7	3	6.3	2.6	36.7	1.5	E	Green	MR
TVu9393	3.5	4.5	5.5	3	3.7	6.3	5.5	42.2	0.5	E	Green	MR
TVu9469	3.0	4.5	5.8	2.8	3.8	6.3	2.7	30.5	2.5	M	Purp	MR
TVNu699	3.3	4.3	5.5	3.3	4.1	6.2	1.6	11.6	2	L	Green	MR
TVu15500	3.3	4.5	5.8	2.5	3.7	5.4	8.7	27.9	1	L	Green	MR
TVu16408	3.0	4.0	5.0	3.0	4.6	6.2	5.3	42.3	0	E	Green	MR
TVu7614	3.5	4.3	5.8	3.2	4.2	6.3	6.3	32.9	0	L	Green	MR
TVu8923	2.8	3.5	5.0	2.7	3.9	6.4	5.2	38.8	0	L	Green	MR
Vita7*	4.3	5.5	6.5	5.1	6.4	7.6	2.5	17.8	2	M	Green	S
F Pr.	<0.001	<0.001	0.05	0.001	<0.001	<0.001	<0.001	<0.001	0.2096			
LSD	1.12	1.2	1.4	1.2	1.8	2.0	3.8	30.5	6.3			

Note

MR: moderate resistant; R: resistant; S: susceptible; thrips pop: population of thrips; *: checks. All the numbers in the table are the means of three replicates; Flow: flowering; E: early flowering (≤45 days); M: medium flowering (46–55 days); L: late flowering (≥56 days)

**Table 2:** Means and heritability values for the main traits measured in 2016 to determine cowpea resistance to *Megalurothrips sjostedti*

Traits	Min	Avg	Max	Heritability
Damage score at 45 DAP	2.7	2.9	3.2	0.111
Damage score at 55 DAP	3.6	4.4	5.2	0.275
Damage score at 65 DAP	4.3	7.2	8.0	0.614
Pods per plant	1.3	2.6	6.4	0.514
Peduncles per plant	7.0	8.7	15.3	0.229
Peduncles with pods	0.8	1.7	3.7	0.502
Flower bud abortion rate	43.7	75.9	90.3	0.590
% Effective peduncles	9.7	24.1	56.3	0.590
Thrips population/flower	1.9	2.4	4.2	0.1556

Note

Avg: average; Max: maximum; Min: minimum.

### 3.2 Pods per plant and % effective peduncles

Test lines with low damage scores showed significantly higher number of pods per plant (ranging from 5.1 to 10.5) except genotypes TVu8877, and TVu16521 with relatively low pod number per plant (3.7 and 4.2, respectively) despite their low damage scores. The highest number of pods per plant among the test entries was produced by TVu9357 (8.7 pods). Test lines with low damage scores showed higher percentage of effective peduncles. The highest percentage of effective peduncles of 78.3% were recorded on TVu7739 (Table 1).

### 3.3 Flowering time and stem/pod colour

The flowering time varied between early, medium and late flowering. Among the resistant and moderately resistant genotypes, twenty‐eight flowered as early as the resistant check Sanzi, fourteen flowered late and twenty including the susceptible check Vita7 were medium flowering (Table 1). With regard to the stem/pod coloration, only eleven genotypes including the resistant Sanzi had purple stem/pods colour (Table1).

### 3.4 Resistance categories

Apart from the four lines that showed good levels of resistance to thrips, fifty‐seven accessions made up of 56 from the mini core collection and one wild relative were regarded as moderately resistant. They had moderate damage scores of between 4.8 and 6.4 and relatively high number of pods (three to five pods/plants) except the wild relative, TVNu699 that had an average of 1.6 pods per plant. Genotypes TVu8877 and TVu16521 despite the low damage scores were placed in this group because of their relatively low pod number per plant (Table 1).

### 3.5 Number of peduncles with pods and flower abortion rate

**Figure 1:**
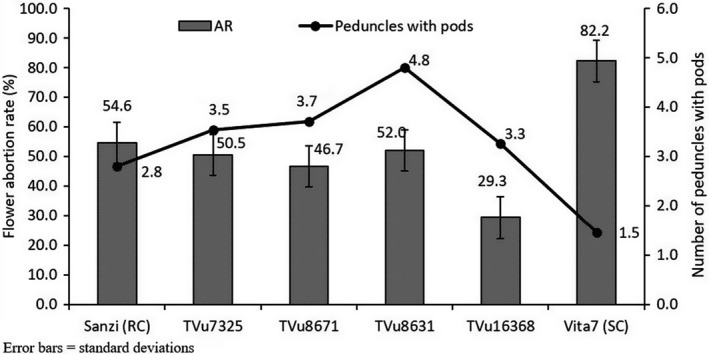
Number of peduncles with pods and flower bud abortion rate (AR) among resistant test lines and checks

Genotypes that had high number of peduncles with pods were characterized by low flower abortion rates. Among the genotypes considered as resistant, the number of peduncles with pods varied from 2.8 (Sanzi) to 5.4 (TVu9357) and the flower abortion rate ranged from 21.7% (TVu7739) to 55.1% (TVu8779). The susceptible line Vita 7 recorded 1.5% and 82.2% for number of peduncles with pods and abortion rate, respectively (Figure 1).

### 3.6 Heritability of measured parameters

Following data analysis using the software Breeding View, it was possible to estimate heritability values for the different measured traits. High levels of heritability were associated with parameters such as damage scores at 65 DAP, pod number per plant, peduncles with pods, flower bud abortion rate and percentage of effective peduncles. The heritability values were comparatively low for damage scores recorded at 45 and 55 DAP and for thrips population in flowers (Table 2).

### 3.7 Relationships between damage scores and other traits

**Figure 2:**
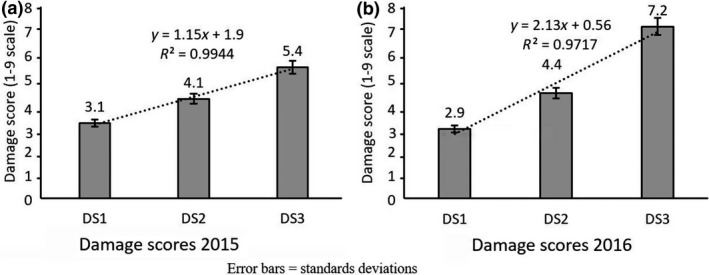
Thrips damage scores (DS1, DS2 & DS3) measures along the reproductive stages of cowpea (45, 55 and 66 days after planting, respectively) in 2015 (a) and 2016 (b)

**Figure 3:**
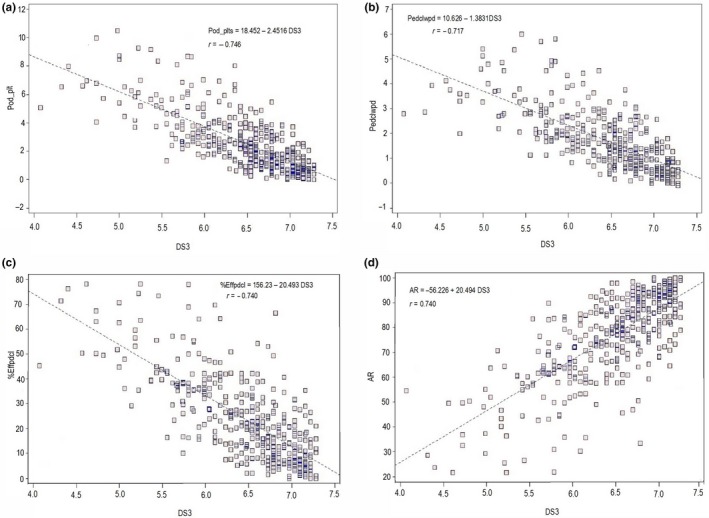
Relationship between thrips damage scores at 65 DAP (DS3) and pods per plant (a), number of peduncles with pods (b), % effective peduncles (c) and flower abortion rates (d) [Colour figure can be viewed at wileyonlinelibrary.com]

The mean values for thrips damage scores (DS) increased linearly from 45 DAP to 65 DAP (Figure 2). Regression analysis showed linear negative relationships between the damage scores at 65 DAP (DS3) and the number of pods per plant (*r* = −0.746), the number of peduncles with pods (*r* = −0.717) and the percentage of effective peduncles (*r* = −0.740). However, a linear positive relationship was obtained between the damage scores at 65 DAP and the flower bud abortion rate (*r* = 0.740; Figure 3).

Similarly, there was a highly significant and positive correlation (*p* < 0.001, *r* = 0.946) between number of pods per plant and number of peduncles with pods. Highly significant and positive correlations were found between damage scores at 45 and 55 DAP (*p* < 0.001; *r* = 0.751), and also between damage scores at 55 and 65 DAP (*p* < 0.001; *r* = 0.732). However, number of pods per plant and damage score values were significantly but negatively correlated at 55 DAP (*p* < 0.05; *r* = −0.518) and 65 DAP (*p* < 0.001; *r* = −0.746; Table 3).

**Table 3:** Estimate genetic correlations between the measured parameters in cowpea genotypes under field evaluation for resistance to *Megalurothrips sjostedti*

Measured traits	1	2	3	4	5	6	7	8
1. Damage score 1	—							
2. Damage score 2	0.751^a^	—						
3. Damage score 3	0.521^b^	0.732^a^	—					
4. Pods per plant	–0.331^ns^	–0.518^b^	–0.746^a^	—				
5. Peduncles per plant	0.240^ns^	0.171^ns^	0.138^ns^	0.071^a^	—			
6. Peduncles with pods	–0.305^ns^	–0.484^ns^	–0.717^a^	0.946^a^	0.111^ns^	—		
7. Abortion rate	0.365^ns^	0.526^b^	0.740^a^	–0.776^a^	0.331^ns^	–0.783^a^	—	
8. %Effective peduncles	–0.365^ns^	–0.526^b^	–0.740^a^	0.776^a^	–0.331^ns^	0.783^a^	−1.000^a^	—

Note

ns: not significant.

* Significant with *p* < 0.05.

*** Highly significant with (*p* < 0.001).

## 4 DISCUSSION

The observed linear progression in thrips damage scores from 45 to 65 DAP in this study, can be attributed to a steady increase in the population density of the insect in the field from flower bud initiation to when the final scores were recorded as earlier suggested by Niassy et al. (2016). Flower bud thrips (*M. sjostedti*) infests cowpea plants from pre‐flowering stage, and because of the insect's short developmental cycle of about 19 days (Salifu, 1992), it is able to produce up to four generations between flower bud initiation and flowering stages of the crop. The higher insect population at 65 DAP resulted in an increased pressure of the insect thereby causing more damage to the plant. Symptoms of thrips attack on cowpea plants are well known. These include browning of stipules and flower buds, several stunted peduncles with no pods as well as flower bud abscission (Jackai & Singh, 1988). Severely infested plants appear diseased and produce only a very limited number of flowers that reach anthesis. This is because most of the flower buds drop prematurely (Singh & Allen, 1979).

Four mini core accessions, TVu8631, TVu16368, TVu8671 and TVu7325, were found to consistently show low thrips damage in 2015 and 2016. These test lines had good pod load and high percentage effective peduncles (≥40.0%) that were similar to the levels shown by the resistant check, “Sanzi.” These lines therefore should be considered as resistant to *M. sjostedti*. Pod load and per cent effective peduncles are key components of cowpea yield and can be considered as traits of interest while assessing the resistance or susceptibility of cowpea genotypes to flower bud thrips. These are traits that breeders can rely upon while making selections for resistant lines in the field.

There were significant differences among the cowpea genotypes for number of peduncles that had pods as well as flower abortion rates. Resistant lines had higher number of peduncles with pods and lower flower abortion rate while the susceptible lines showed opposite tendencies. As expected, the damage scores recorded at 65 DAP were significantly and negatively correlated with many of the yield‐related traits such as the number of pods per plant and the percentage of effective peduncles. This implies that thrips damage scores at the late flowering stage (65 DAP) are more reliable when assessing the resistance status of cowpea genotypes to *M. sjostedti *than the scores at 45 and 55 DAP. A positive correlation was observed between flower abortion rate and damage scores at 65 DAP. The number of peduncles with pods and flower abortion rates can also be considered as key traits in assessing for resistance of cowpea to *M. sjostedti* in addition to the damage scores at the late flowering stage, pod load and percentage effective peduncles. Usually, peduncles with pods are elongated compared to the non‐effective type as observed in our study. We did not find any significant difference in thrips populations in flowers among the test entries; however, we observed that the damage recorded on the susceptible lines were higher than that on resistant ones. As shown in Table 2, there is a relationship between insect population in flowers and damages scores. This result corroborates that of Salifu, Hodgson, and Singh (1988), who found heavy infestation of thrips on susceptible lines at the flower bud stage leading to complete abortion of the flowers.

In this study, 57 accessions were identified as moderately resistant to *M. sjostedti *based on their moderate damage scores (which ranged from 4.5 to 6.3) and their average pod number per plant (three to five pods). Jackai and Singh (1988) following an earlier study had suggested that cowpea lines with damage scores of between five and six should be considered as moderately resistant to thrips. The mechanism of resistance operating in these genotypes could be a tolerance because they were able to produce some pods despite the fairly high levels of thrips damage observed on them in the field.

In this study, the resistant and moderately resistant genotypes displayed some characters such as high pod load and effective peduncles. These parameters are yield‐related traits resulting from low flower abortion in thrips resistant/tolerant cultivars. According to Togola et al. (2017), cowpea cultivars with ability to overcome or compensate damage caused by insect pest can produce more pods. Of the 61 resistant and moderately resistant, 28 were early flowering type and 14 were late flowering cultivars. According to Asante, Tamo, and Jackai (2001), early and late flowering are plant phenological traits that can allow cultivars to escape pest damage. The purple coloration of stem or pod shown in some cultivars might result in anthocyanin pigments or flavonoid content (Yoshida, Oyama, & Kondo, 2012) reported to confer antibiosis resistance to insect pests (Dabire‐Binso, Ba, Sanon, Drabo, & Bi, 2010; Lattanzio, Arpaia, Cardinali, Venere, & Linsalata, 2000). Further studies are necessary to confirm the exact mechanism present in each of the identified resistant cowpea lines.

The heritability values for some of the measured traits such as the damage scores at 65 DAP, the number of pods per plant, the number of peduncles with pods and the percentage of effective peduncles were high (>50%), implying that these traits are heritable and environmental effects on them are relatively low. Breeders can therefore make progress while developing improved cowpea varieties with resistance to flower thrips by using these traits as selection criteria. This is particularly so, when selection is based on low damage scores, low flower abortion rate and high number of effective peduncles. The results of this study show the superiority of the four resistant genotypes which can be as good as Sanzi, an already identified source of thrips resistance in cowpea. These four resistant genotypes should be further evaluated to ascertain the types of genes coding for their resistance. Data from the genetic studies will enable breeders to effectively harness the resistance gene(s) for the development of improved cowpea varieties that are resistant to flower bud thrips.

## 5 CONCLUSION

The need for the identification of sources of resistance to *M. sjostedti *in cowpea has remained a front burner in cowpea breeding programs in SSA. With the identification of several promising lines characterized by good levels of resistance to flower bud thrips, development of improved cowpea with resistance to this pest appears feasible in the foreseeable future. It is conceivable that different gene loci could be responsible for the resistance detected among some of the 4 lines in this study. It will therefore be necessary to determine if allelism exists among some or all of the resistant lines. From this study, those lines not showing allelism could be crossed for the purpose of pyramiding the genes present in the development of thrips resistant varieties. This will ensure a robust and more durable resistance in improved varieties containing the pyramided genes. The high number of genes will serve as buffer in controlling thrips damage in the new improved varieties.

## AUTHORS' CONTRIBUTIONS

Togola Abou, Boukar Ousmane, Chamarthi Siva, and Fatokun Christian conceived research, analysed data, conducted statistical analyses and interpreted the results. Belko Nouhoun, Tamò Manuele and Oigiangbe Nathaniel contributed in drafting the manuscript and revising it critically for important intellectual content. Ojo Joseph and Ibikunle Mumini conducted experiments, collected data in the field.
